# Unconventional Recombination in the Mating Type Locus of Heterothallic Apple Canker Pathogen *Valsa mali*

**DOI:** 10.1534/g3.116.037853

**Published:** 2017-02-21

**Authors:** Zhiyuan Yin, Xiwang Ke, Zhengpeng Li, Jiliang Chen, Xiaoning Gao, Lili Huang

**Affiliations:** State Key Laboratory of Crop Stress Biology for Arid Areas, College of Plant Protection, Northwest A&F University, Yangling, Shaanxi 712100, China

**Keywords:** *Cytospora* sp., sexual reproduction, *MAT*, unequal recombination

## Abstract

Sexual reproduction in filamentous ascomycetes is controlled by the mating type (*MAT*) locus, including two idiomorphs *MAT1-1* and *MAT1-2*. Understanding the *MAT* locus can provide clues for unveiling the sexual development and virulence factors for fungal pathogens. The genus *Valsa* (Sordariomycetes, Diaporthales) contains many tree pathogens responsible for destructive canker diseases. The sexual stage of these ascomycetes is occasionally observed in nature, and no *MAT* locus has been reported to date. Here, we identified the *MAT* locus of the apple canker pathogen *Valsa mali*, which causes extensive damage, and even death, to trees. *V. mali* is heterothallic in that each isolate carries either the *MAT1-1* or *MAT1-2* idiomorph. However, the *MAT* structure is distinct from that of many other heterothallic fungi in the Sordariomycetes. Two flanking genes, *COX13* and *APN2*, were coopted into the *MAT* locus, possibly by intrachromosomal rearrangement. After the acquisition of foreign genes, unequal recombination occurred between *MAT1-1/2* idiomorphs, resulting in a reverse insertion in the *MAT1-2* idiomorph. Evolutionary analysis showed that the three complete *MAT1-1-2*, *COX13*, and *APN2* genes in this region diverged independently due to different selection pressure. Null hypothesis tests of a 1:1 *MAT* ratio of 86 *V. mali* isolates from four different provinces showed a relatively balanced distribution of the two idiomorphs in the fields. These results provide insights into the evolution of the mating systems in Sordariomycetes.

Sexual reproduction is important in pathogenic ascomycetes because new combinations of virulence alleles are created through outcrossing (McDonald and Linde 2002). Mating in filamentous ascomycetes is typically controlled by a single locus, termed the mating-type (*MAT*) locus, which includes two alleles called *MAT1-1* and *MAT1-2*. These alleles have been termed “idiomorphs” due to their high sequence divergence (Metzenberg and Glass 1990). Filamentous ascomycetes generally exhibit self-incompatible (heterothallic) or self-compatible (homothallic) lifestyles. In heterothallic ascomycete fungi, the *MAT* locus carries either the *MAT1-1* or the *MAT 1-2* idiomorph, which contains at least a *MAT1-1-1* α-domain gene or a *MAT1-2-1* high-mobility-group (HMG) domain gene, respectively. In addition, several additional genes are also found in various ascomycetes, although the functions of these genes remain obscure (Dyer *et al.* 2016). These mating-type genes function not only in controlling sexual development, but also in regulating fungal secondary metabolites and hyphal morphology (Kück and Böhm 2013). Thus, understanding the *MAT* locus can provide insights into the sexual development and virulence factors in filamentous ascomycetes.

The genus *Valsa* (Sordariomycetes, Diaporthales) contains >500 species, including many aggressive tree pathogens responsible for canker diseases. *Valsa* cankers affect >70 species of trees worldwide, and often cause extensive damage to trees (Agrios 2005). *V. mali* Miyabe et Yamada [anamorph *Cytospora sacculus* (Schwein.) Gvrit.], causing destructive canker on apple and resulting in severe yield losses in eastern Asia (Lee *et al.* 2006; Li *et al.* 2013), infects mainly apple by conidia, while ascospores are not common (Wang *et al.* 2014; Wang *et al.* 2016) (Figure 1). Of the 150 infected apple bark samples from different regions in China, only eight from dead trunks have ascostromata with ascospores (Wang 2007). In addition, the sexual reproduction of *Valsa* spp. cannot be induced in the laboratory to date, and no *MAT* locus in *Valsa* has been reported. Thus, in this study, we identified and delineated the structure of the *MAT* locus of *V. mali* using comparative genomic approaches, and investigated the evolution of *MAT* genes and loci in *V. mali* and closely related species.

**Figure 1:**
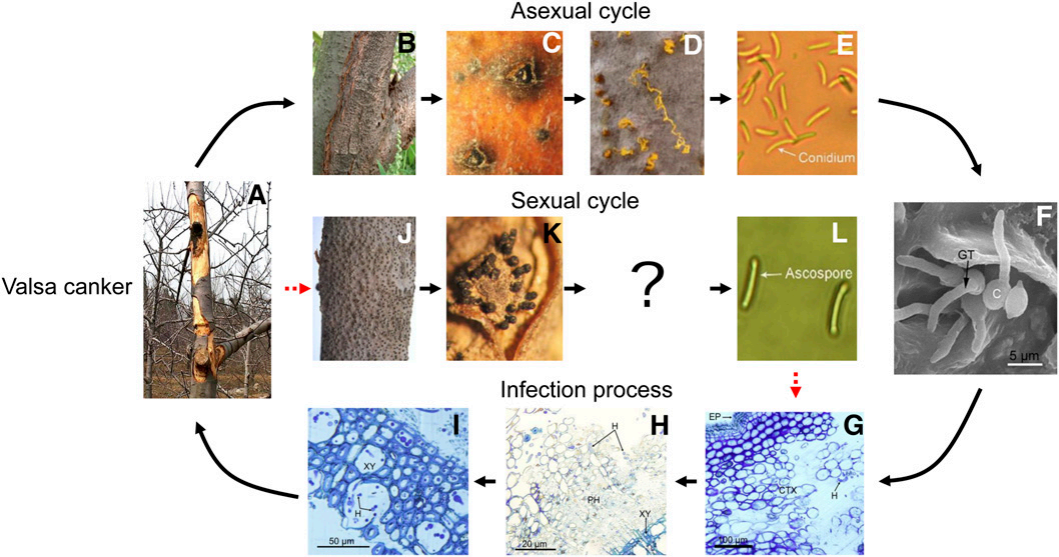
(A–L) Disease cycle of Valsa canker caused by *V. mali*. (A) Lesions on apple bark caused by *V. mali*. The infected bark is scraped. (B) Conidiomata formed in the canker. (C) Pycnidia. (D) Discharged conidia. (E) Conidium. (F) Conidial germination. (G–I) Light micrographs of infected bark. (J) Ascostromata formed in the canker. (K) Ascostromata. (I) Ascospore. The pathogen infects mainly apple by conidia, and sexual reproduction is occasionally observed in nature. Successful infection often occurs on wounded tissues, and infection hyphae develop mainly in the cortex and phloem. C, conidia; CTX, cortex; EP, epidermis; GT, germ tube; H, hyphae; PH, phloem; XY, xylem.

## Materials and Methods

### Strains and culture conditions

All strains used in this study were deposited at the Laboratory of Integrated Management of Plant Diseases in College of Plant Protection, Northwest A&F University, Yangling, PR China. Cultures were grown on potato dextrose agar (PDA) medium with a layer of cellophane at 25°.

### Identification of the V. mali MAT locus

The mating type locus of *V. mali* strain 03-8 was identified by BLASTP searches against *V. mali* proteome (>1E−5) using protein sequences of *MAT1-1-1* and *MAT1-2-1* from the closely related species *Cryphonectria parasitica* as query sequences, which suggests that *V. mali* is heterothallic, and that strain 03-8 carries the *MAT1-1* idiomorph. *MAT1-2* candidate isolates were then identified by PCR using a *VmMAT1-1-1* specific primer pair. To identify the *MAT1-2* idiomorph, the genome of isolate SXLC146 was sequenced using Illumina HiSeq technology. Filtered paired-end reads were assembled by ABySS v1.9.0 (Simpson *et al.* 2009), and gene models were predicted using MAKER v2.31.8 (Holt and Yandell 2011). Primer pairs of *VmMAT1-1-1* (F: 5′-GAAAGGTCGGAAAGGCAAAG-3′ and R: 5′-AGAGTCGGGTCGGGCAAT-3′), and *VmMAT1-2-1* (F: 5′-CAACATTGGCATTCAACTCA-3′ and R: 5′-CTTGCTTCGTCGCTTCAC-3′), were used for PCR detection of isolates from different geographic regions.

### Evolutionary analyses of the MAT locus

Synteny of the *MAT* locus between *MAT1-1* and *MAT1-2* idiomorphs was analyzed using GATA (Nix and Eisen 2005). Protein sequences of mating type genes were aligned using MAFFT v7.245 (Katoh and Standley 2013), and poorly aligned regions were removed by trimAl v1.4 (Capella-Gutiérrez *et al.* 2009). Maximum likelihood trees were constructed by IQtree v1.3.11 (Nguyen *et al.* 2015), using the build-in best evolutionary model selection function. Branch supports were assessed with ultrafast bootstrap method (Minh *et al.* 2013) and SH-aLRT test (1000 replicates). Selection pressure on mating type genes were tested at the codon level using the ete evol tool in ETE package v3.0 (Huerta-Cepas *et al.* 2016). The coding sequence alignments of these genes were constructed by the ETE package using several build-in alignment tools, and CodeML and Slr analyses were then performed by the ete-evol program. Sites under selection were identified using the M2 and SLR models. The null hypothesis of a 1:1 *MAT* ratio of *V. mali* was tested using chi-square goodness-of-fit test using the online tool VassarStats (http://vassarstats.net/).

### Transmission electron microscopy (TEM)

The perithecium, ascus, and ascospore of *V. mali* in the field were investigated by TEM. Ascostromata samples from the canker were processed for TEM as described by Ke *et al.* (2013). For TEM, ultrathin sections of specimens cut with a diamond knife were collected on copper grids. After contrasting with uranyl acetate and lead citrate, the grids were examined with a TEM 1230 (JEOL) at 80 kV.

### Data availability

All strains used in this study are available upon request. The raw Illumina reads of isolate SXLC146 have been deposited at the Sequence Read Archive (SRA) database of NCBI (SRP075864). The nucleotide sequence of *MAT1-2* idiomorph has been deposited at the GenBank database (KX349090).

## Results and Discussion

### Identification of MAT1-1 idiomorph

**Figure 2:**
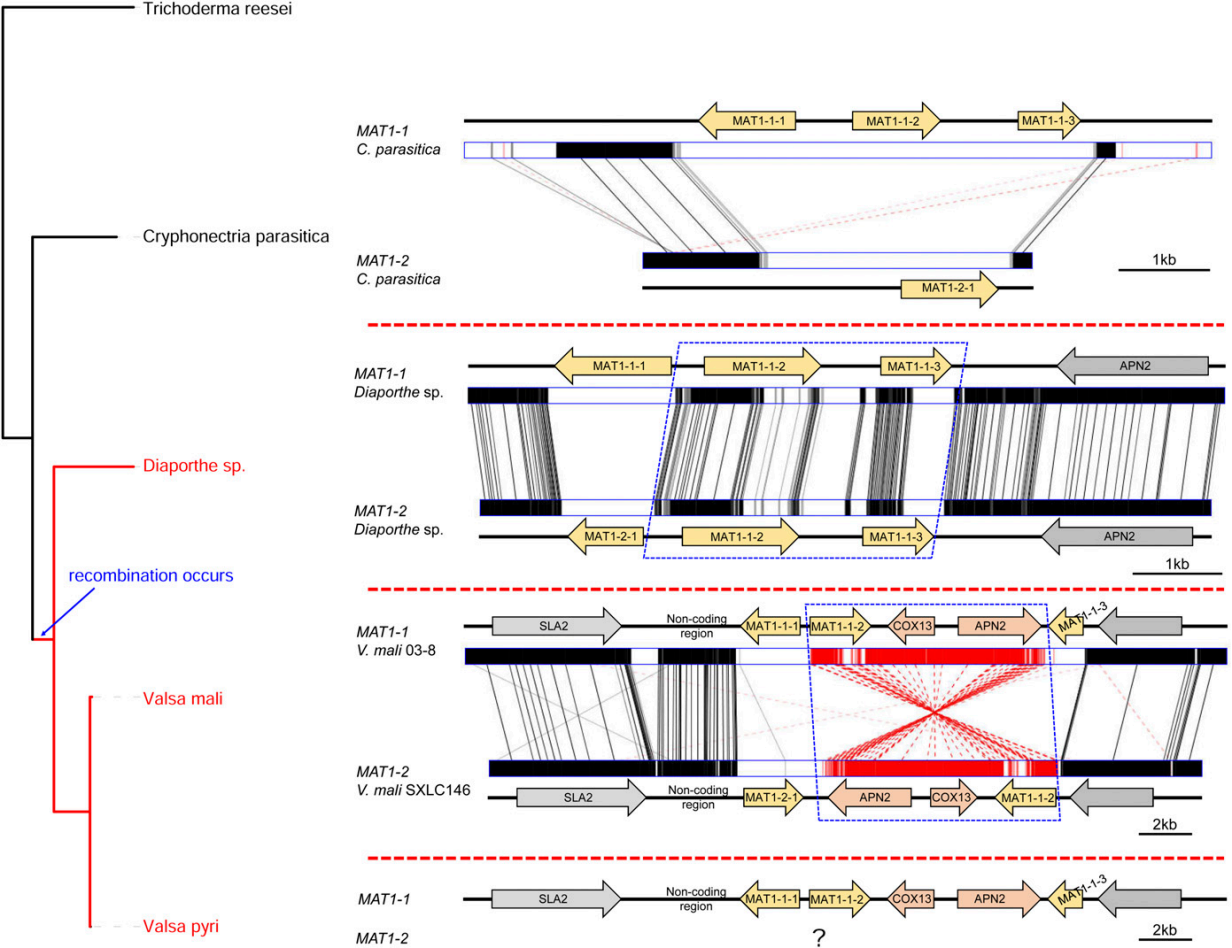
Mating type idiomorphs of *Valsa* spp. and closely related species. The phylogenetic tree on the left side was constructed on the basis of *MAT1-1-1*. Sequences of *C. parasitica* and *Diaporthe* spp. were retrieved from GenBank according to McGuire *et al.* (2001) and Kanematsu *et al.* (2007), respectively. The blue dashed box indicates recombination region.

To identify the mating type locus in *V. mali*, protein sequences of core mating type genes *MAT1-1-1* (GenBank: AAK83346) and *MAT1-2-1* (AAK83343) of the closely related species *C. parasitica* were used to search against the genome of *V. mali* strain 03-8 (GCA_000818155.1) (Yin *et al.* 2015). By performing BLASTP searches, only *MAT1-1-1* was found in strain 03-8. The absence of the *MAT1-2-1* HMG-box gene in the *MAT* locus of strain 03-8 suggests that *V. mali* is likely heterothallic. *VmMAT1-1-1* (VM1G_08160) contains the typical α-domains, and adjacent genes include *SLA2* (VM1G_08159), *MAT1-1-2* (VM1G_08161), *COX13* (VM1G_08162), *APN2* (VM1G_08163), and *MAT1-1-3* (VM1G_08164) (Figure 2). Intriguingly, *COX13* and *APN2* locate in the *MAT* locus, while the location of *SLA2* and *APN2* is fairly conserved, and often flanks the idiomorph among other mating type genes in many other filamentous ascomycetes (Dyer *et al.* 2016), such as *C*. *parasitica* (McGuire *et al.* 2001). Likewise, idiomorphs of two heterothallic species *Coccidioides immitis* (Fraser *et al.* 2007) and *Uncinocarpus reesii* (Mandel *et al.* 2007) also captured *COX13* and *APN2* into the *MAT* locus, while both these genes are adjacent to the *MAT* locus in closely related species. However, the role or influence of this kind of remodeling remains unknown.

As a heterothallic species, there must be *MAT1-2* isolates of *V. mali*. However, *MAT1-1* and *MAT1-2* isolates of filamentous ascomycetes are morphologically indistinguishable for most of their life cycles (Debuchy *et al.* 2010). To identify *MAT1-2* isolates, a specific primer pair for *VmMAT1-1-1* (F: 5′-GAAAGGTCGGAAAGGCAAAG-3′ and R: 5′-AGAGTCGGGTCGGGCAAT-3′) was used to detect *V. mali* isolates. Isolate SXLC146, without a PCR product, was then used to identify the *MAT1-2* idiomorph.

### Identification of the MAT1-2 idiomorph

To determine the structure of the *MAT1-2* idiomorph, the genome of isolate SXLC146 was sequenced *de novo* by Illumina HiSeq-PE150 platform. A total of 20,860,413 clean reads (5.2G, effective rate 96.46%) were subjected to assembly. Nucleotide sequences of genes flanking the *VmMAT1-1* idiomorph were used to perform BLASTN searches against genome assemblies of isolate SXLC146. Gene models of the scaffold that contains the *MAT1-2* idiomorph were then predicted using MAKER v2.31.8 (Holt and Yandell 2011). *VmMAT1-2-1* (GenBank: KX349090) contains the HMG-box, and adjacent genes includes *SLA2*, *APN2*, *COX13*, and *MAT1-1-2* (Figure 2). Similar to the *VmMAT1-1* idiomorph, the *VmMAT1-2* idiomorph also captured *APN2* and *COX13* into the *MAT* locus. The *MAT* locus organization indicates that *V. mali* is heterothallic. Unexpectedly, a mating type gene *MAT1-1-2* of the *MAT1-1* idiomorph is present in the *MAT1-2* idiomorph. *MAT1-1-2* is ubiquitous in Sordariomycetes, and is required for fruit body development (Debuchy *et al.* 2010). However, the involvement of *MAT1-1-2* (especially in the *MAT1-2* idiomorph) in sexual development of *V. mali* remains unknown.

In order to test the null hypothesis of a 1:1 *MAT* ratio of *V. mali*, 86 isolates from four different provinces were detection by PCR using two pairs of specific primers targeting *VmMAT1-1-1* and *VmMAT1-2-1*, respectively (Supplemental Material, Table S1). Both idiomorphs were present in the four provinces, and their *MAT* ratios did not deviate significantly from 1:1 (Table 1), which suggests a relatively balanced distribution of mating-type idiomorphs in the fields.

**Table 1:** *MAT1-1*/*MAT1-2* ratio tests on populations of *V. mali*

Population	Total Number	*MAT1-1*	*MAT1-2*	χ^2^, *P* Value
Baoji, Shaanxi	10	4	6	0.4, *P* = 0.7518
Yuncheng, Shanxi	24	9	15	1.5, *P* = 0.3078
Tianshui, Gansu	13	6	7	0.08, *P* = 1
Sanmenxia, Henan	30	12	18	1.2, *P* = 0.3594
All isolates	86	35	51	2.98, *P* = 0.1055

### Unconventional recombination of MAT locus in V. mali

Recombination at the *MAT* locus in ascomycetes is thought to be suppressed (Idnurm 2011). However, synteny analysis showed that the region carrying *APN2*, *COX13*, and *MAT1-1-2* in the *MAT1-2* idiomorph is a reverse insertion, probably acquired from the *MAT1-1* idiomorph by recombination (Figure 2). Additionally, *MAT1-1-2* contains many more sequence variations than *APN2* and *COX13*. Protein sequence identity of these three genes are 100% (*COX13*), 92.13% (*APN2*), and 76.89% (*MAT1-1-2*), respectively. A similar event was also reported in the closely related species *Diaporthe* spp., the *MAT1-2* idiomorph of which carries homologs of *MAT1-1-2* (identity: 80.95%) and *MAT1-1-3* (54.62%) in the same gene order and orientation as that in *MAT1-1* (Kanematsu *et al.* 2007) (Figure 2). These unconserved “additional” mating type genes, caused by unequal recombination, are probably also functional, because they are transcriptionally active during vegetative growth (Kanematsu *et al.* 2007). Nevertheless, future work is required to determine the functions of these genes in sexual reproduction.

Unequal recombination at the *MAT* locus has also been demonstrated in many other ascomycetes, but genes involved in those events are often fragments or truncated pseudogenes (Tsui *et al.* 2013). In *V. mali*, unequal recombination resulted in the presence of three compete genes in *MAT1-2*. The types of *MAT* structure in *Valsa* spp. (including *V. malicola*, *V. sordida*, and *V. persoonii*, Z. Yin and L. Huang, unpublished results) and *Diaporthe* spp. are unconventional and distinct from known heterothallic filamentous ascomycetes. In addition, another closely related heterothallic species, *C. parasitica*, which causes chestnut blight, has the typical *MAT* structure in heterothallic Sordariomycetes, carrying three genes, *MAT1-1-1*, *MAT1-1-2*, and *MAT1-1-3* in the *MAT1-1* idiomorph and only one gene, *MAT1-2-1*, in the *MAT1-2* idiomorph (McGuire *et al.* 2001) (Figure 2). This finding suggests that this unconventional recombination occurred evolutionally from specific families in Diaporthales. Given that homothallism has likely evolved from heterothallic ascomycetes (Billiard *et al.* 2011; Dyer *et al.* 2016), the *MAT* structures of *V. mali* and *Diaporthe* spp. will provide clues for unveiling the evolutionary history of the mating systems in Sordariomycetes. However, to confirm this hypothesis, it is necessary to determine the *MAT* loci of additional species (especially homothallic) in Diaporthales.

### Evolutionary analyses of mating type genes

**Figure 3:**
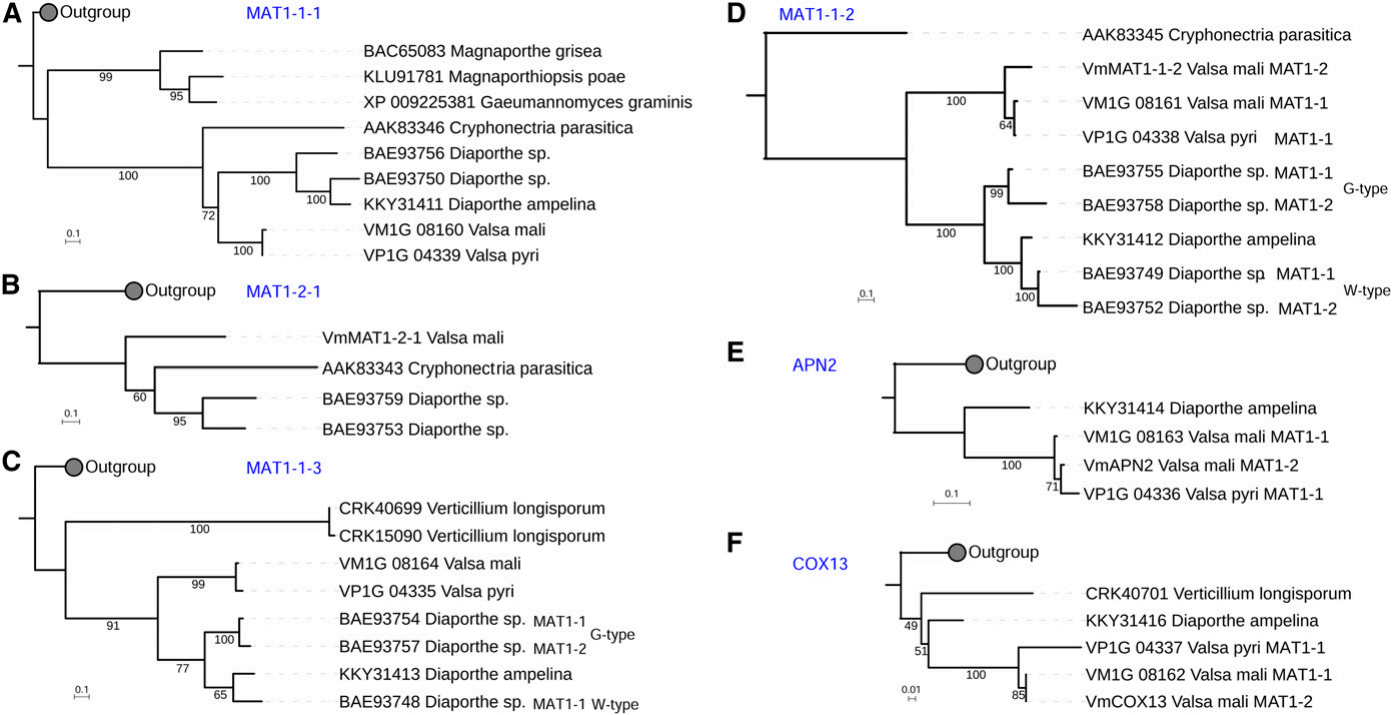
Phylogeny of mating type genes: (A) *MAT1-1-1*, (B) *MAT1-2-1*, (C) *MAT1-1-3*, (D) *MAT1-1-2*, (E) *APN2*, (F) *COX13*. Maximum likelihood phylogenetic trees were constructed from top 20 BLASTP hits in GenBank using IQtree. The scale bar represents substitutions per site.

The same *MAT* structure in *V. mali* and *V. pyri*, as well as the scenario in *Diaporthe* spp., indicates that recombination predates speciation (Figure 2). The three foreign genes (*APN2*, *COX13*, and *MAT1-1-2*) in the *MAT1-2* idiomorph were unlikely to have been acquired independently, and probably diverged independently. We were thus interested in the phylogeny of these genes. For each mating type gene of *V. mali*, the protein sequences of the top 20 blast hits in the GenBank nr database were aligned, and subjected to maximum likelihood phylogenetic tree construction. The tree of the *COX13* gene shows that this gene in the *VmMAT1-2* idiomorph is more closely related to the homolog in *VmMAT1-1* than to that in the *MAT1-1* idiomorph of *V. pyri* (Figure 3), which suggests that *COX13* was acquired before the divergence of *Valsa* species. Likewise, the *MAT1-1-2* and *MAT1-1-3* genes are present in both idiomorphs of the *Diaporthe* sp. group within G-type and W-type species, respectively. The *VmCOX13* genes are highly conserved in both idiomorphs, and ancestral *VmAPN2* and *VmMAT1-1-2* genes are thus likely to have the same phylogenetic relationship as *VmCOX13*. However, the *VmMAT1-1-1* gene in *MAT1-1* is closely related to that in *MAT1-1* of *V. pyri*, while *VmAPN2* in *MAT1-2* groups with that in *MAT1-1* of *V. pyri* (Figure 3). This result suggests that the *APN2*, *COX13*, and *MAT1-1-2* genes in the different idiomorphs diverged independently after acquisition.

**Figure 4:**
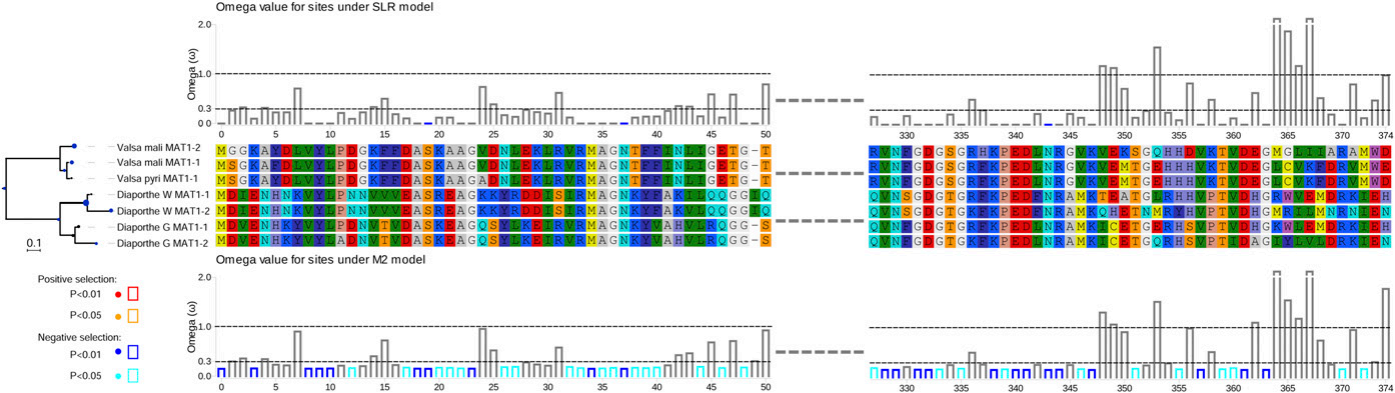
Evolutionary analysis of *MAT1-1-2*. The CodeML and Slr analyses were performed using the ete evol tool in ETE package. Site models M2 and SLR, and branch model fb, respectively, were used. Omega value of branch is represented in the node size and color. Small blue disk on the node of phylogenetic tree stands for low omega value.

**Figure 5:**
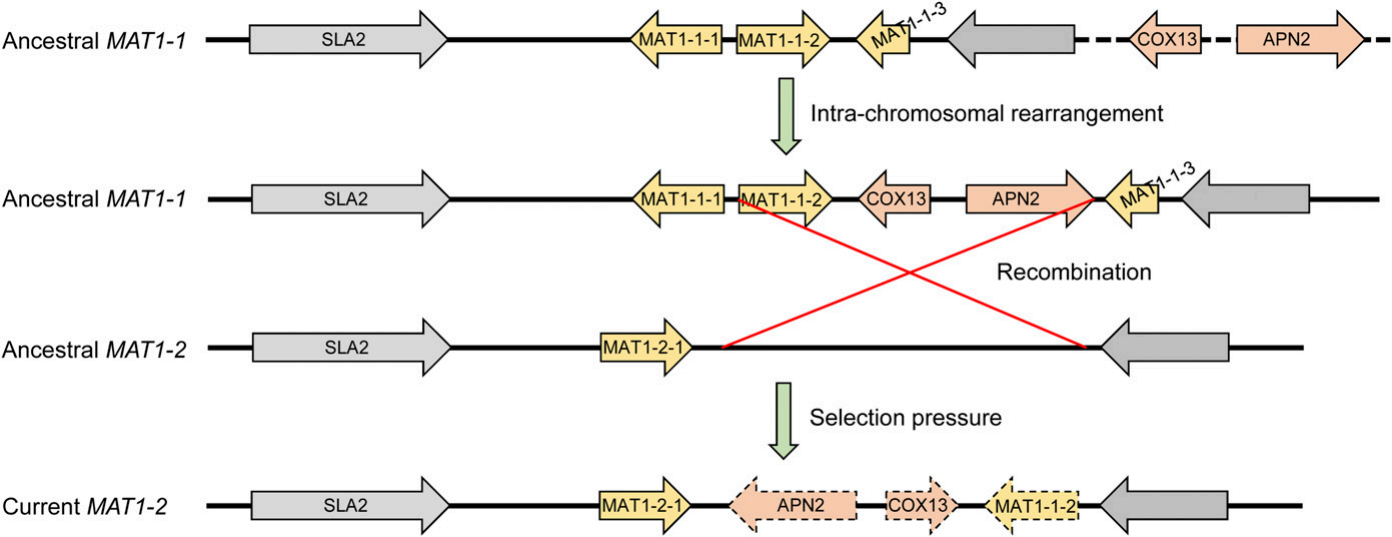
Proposed evolution scenario of *MAT* locus in the ancestor of *Valsa mali*.

Selection pressure analysis of the three acquired genes showed that *MAT1-1-2* has been under purifying selection at interspecific level, indicating that this gene is preserved for proper function (Figure 4). Many sites of *MAT1-1-2* under purifying selection are likely responsible for the dramatically divergence. In contrast, one site (60E) in the nuclease domain of *APN2* is under positive selection, while the *COX13* gene may go through neutral evolution without any site under positive or purifying selection. Thus, we can speculate that the different selection pressure of these three genes results in different levels of sequence divergence. Collectively, a possible scenario of the evolution of *MAT* loci in *Valsa* spp. is that ancestral *MAT1-1* first coopted *APN2* and *COX13* into the *MAT* locus by intrachromosomal rearrangement, and the ancestral *MAT1-2* then acquired *MAT1-1-2*, *COX13* and *APN2* by unequal recombination; finally these three genes diverged independently due to different selection pressure (Figure 5).

### Cryptic sexuality

**Figure 6:**
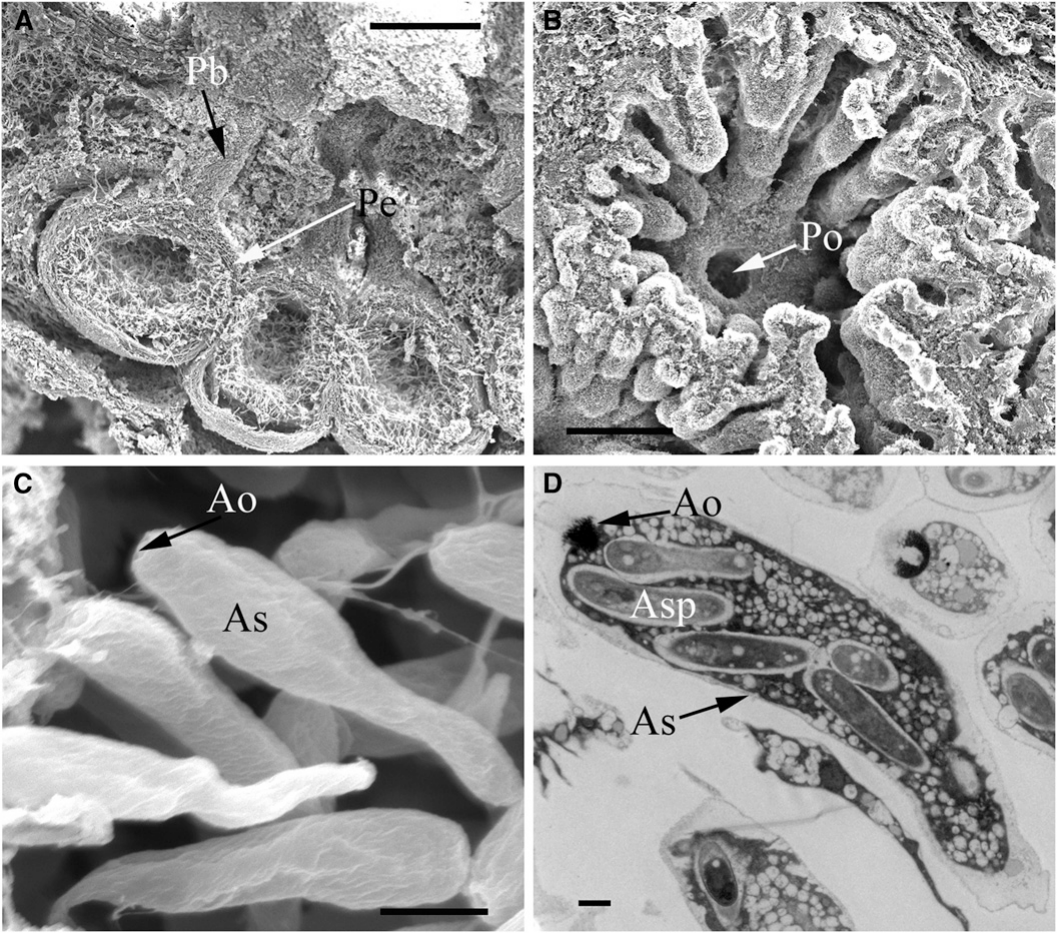
Scanning electron micrographs and TEM graphs of the perithecium, ascus, and ascospore of *V. mali*. (A) longitudinal section of the perithecium (Pe) with a single cavity and beak (Pb) under apple bark. (B) Transverse section of perithecium (Pe) showed a single orifice (Po) on the perithecial beak. (C) Clavate ascus (As) in perithecium with ascus orifice (Ao). (D) TEM graph of asci (As) with ascospores (Asp). Bars, (A) 200 μm; (B) 200 μm; (C) 5 μm; (D) 1 μm.

In nature, sexual reproduction of *V. mali* is occasionally observed, often on dead apple trunks in autumn. The matured ascostromata could be identified on apple barks by the exposed surface, which has many minute black papillae (Figure 1J). Each papilla has an opening of a long neck connected with a perithecium. A longitudinal section of the ascostroma shows that spherical or subglobose perithecia are partly immersed in bark (Figure 6A). When fully matured, the cavity of the perithecium is closely packed with eight asci, which are formed from the basal inner wall of the perithecium. The asci are clavate-oblong or clavate-fusiform, rounded or truncate at the apex, and subsessile (Figure 6C). Ascospores are produced in sausage-shaped asci (Figure 6D) (Ideta 1909; Wang *et al.* 2011), and discharged into the air during wet weather (Agrios 2005).

Attempts to induce self-fertilization of *V. mali* in the laboratory failed. Mycelial plugs of two isolates with opposite mating types (03-8 and SXLC146) were placed at opposite sides of a sterile apple twig embedded on agar according to successful mating tests in *C. parasitica* (Marra and Milgroom 2001) and *Diaporthe* spp. (Kanematsu *et al.* 2000). However, selfing of *C*. *parasitica* and *Diaporthe* spp. is still a rare event in the laboratory, in that only some cross assays succeed (Kanematsu *et al.* 2000; Marra and Milgroom 2001). In addition, mature perithecia were more likely to develop successfully in crosses between isolates derived from a single ascus naturally formed on host twigs, compared to a randomly selected ascus (Kanematsu *et al.* 2000). Therefore, it is necessary to test more isolates of *V. mali*, especially isolates from the same ascus.

## Supplementary Material

Supplemental material is available online at www.g3journal.org/lookup/suppl/doi:10.1534/g3.116.037853/-/DC1.

## References

[bib1] Agrios G., 2005 Plant Pathology. Academic Press, New York.

[bib2] Billiard S. López-Villavicencio M. Devier B. Hood M. E. Fairhead C., 2011 Having sex, yes, but with whom? Inferences from fungi on the evolution of anisogamy and mating types. Biol. Rev. Camb. Philos. Soc. 86: 421–442. 21489122 10.1111/j.1469-185X.2010.00153.x

[bib3] Capella-Gutiérrez S. Silla-Martínez J. M. Gabaldón T., 2009 trimAl: a tool for automated alignment trimming in large-scale phylogenetic analyses. Bioinformatics 25: 1972–1973. 19505945 10.1093/bioinformatics/btp348 PMC2712344

[bib4] Debuchy R. Berteaux-Lecellier V. Silar P., 2010 Mating systems and sexual morphogenesis in Ascomycetes, pp. 501–535 in Cellular and Molecular Biology of Filamentous Fungi, edited by Borkovich K. A. Ebbole D. J. American Society of Microbiology, Washington, DC.

[bib5] Dyer P. Inderbitzin P. Debuchy R., 2016 Mating-type structure, function, regulation and evolution in the Pezizomycotina, pp. 351–385 in *Growth*, *Differentiation and Sexuality*, edited by Wendland J. Springer, New York.

[bib6] Fraser J. A. Stajich J. E. Tarcha E. J. Cole G. T. Inglis D. O., 2007 Evolution of the mating type locus: insights gained from the dimorphic primary fungal pathogens *Histoplasma capsulatum*, *Coccidioides immitis*, and *Coccidioides posadasii*. Eukaryot. Cell 6: 622–629. 17337636 10.1128/EC.00018-07 PMC1865663

[bib7] Holt C. Yandell M., 2011 MAKER2: an annotation pipeline and genome-database management tool for second-generation genome projects. BMC Bioinformatics 12: 491. 22192575 10.1186/1471-2105-12-491 PMC3280279

[bib8] Huerta-Cepas J. Serra F. Bork P., 2016 ETE 3: reconstruction, analysis, and visualization of phylogenomic data. Mol. Biol. Evol. 33: 1635–1638. 26921390 10.1093/molbev/msw046 PMC4868116

[bib9] Ideta A., 1909 *Handbook of the Plant Disease of Japan*, Ed. 4 Shokwabo, Tokyo.

[bib10] Idnurm A., 2011 Sex and speciation: the paradox that non-recombining DNA promotes recombination. Fungal Biol. Rev. 25: 121–127. 23136582 10.1016/j.fbr.2011.07.003 PMC3489284

[bib11] Kanematsu S. Minaka N. Kobayashi T. Akira K. Ohtsu Y., 2000 Molecular phylogenetic analysis of ribosomal DNA internal transcribed spacer regions and comparison of fertility in *Phomopsis* isolates from fruit trees. J. Gen. Plant Pathol. 66: 191–201.

[bib12] Kanematsu S. Adachi Y. Ito T., 2007 Mating-type loci of heterothallic *Diaporthe* spp.: homologous genes are present in opposite mating-types. Curr. Genet. 52: 11–22. 17476509 10.1007/s00294-007-0132-3

[bib13] Katoh K. Standley D. M., 2013 MAFFT multiple sequence alignment software version 7: improvements in performance and usability. Mol. Biol. Evol. 30: 772–780. 23329690 10.1093/molbev/mst010 PMC3603318

[bib32] Ke X. Huang L. Han Q. Gao X. Kang Z., 2013 Histological and cytological investigations of the infection and colonization of apple bark by *Valsa mali* var. *mali*. Australasian Plant Pathol. 42: 85–93.

[bib14] Kück U. Böhm J., 2013 Mating type genes and cryptic sexuality as tools for genetically manipulating industrial molds. Appl. Microbiol. Biotechnol. 97: 9609–9620. 24085397 10.1007/s00253-013-5268-0

[bib15] Lee D. H. Lee S. W. Choi K. H. Kim D. A. Uhm J. Y., 2006 Survey on the occurrence of apple diseases in Korea from 1992 to 2000. Plant Pathol. J. 22: 375–380.

[bib16] Li Z. Gao X. Du Z. Hu Y. Kang Z., 2013 Survey of apple Valsa canker in Weibei area of Shaanxi Province. Acta Agric. Boreali Occidentalis Sin. 1: 029.

[bib17] Mandel M. A. Barker B. M. Kroken S. Rounsley S. D. Orbach M. J., 2007 Genomic and population analyses of the mating type loci in *Coccidioides* species reveal evidence for sexual reproduction and gene acquisition. Eukaryot. Cell 6: 1189–1199. 17513566 10.1128/EC.00117-07 PMC1951113

[bib18] Marra R. E. Milgroom M. G., 2001 The mating system of the fungus *Cryphonectria parasitica*: selfing and self-incompatibility. Heredity 86: 134–143. 11380658 10.1046/j.1365-2540.2001.00784.x

[bib19] McDonald B. A. Linde C., 2002 Pathogen population genetics, evolutionary potential, and durable resistance. Annu. Rev. Phytopathol. 40: 349–379. 12147764 10.1146/annurev.phyto.40.120501.101443

[bib20] McGuire I. C. Marra R. E. Turgeon B. G. Milgroom M. G., 2001 Analysis of mating-type genes in the chestnut blight fungus, *Cryphonectria parasitica*. Fungal Genet. Biol. 34: 131–144. 11686678 10.1006/fgbi.2001.1295

[bib21] Metzenberg R. L. Glass N. L., 1990 Mating type and mating strategies in *Neurospora*. Bioessays 12: 53–59. 2140508 10.1002/bies.950120202

[bib22] Minh B. Q. Nguyen M. A. T. von Haeseler A., 2013 Ultrafast approximation for phylogenetic bootstrap. Mol. Biol. Evol. 30: 1188–1195. 23418397 10.1093/molbev/mst024 PMC3670741

[bib23] Nguyen L. T. Schmidt H. A. von Haeseler A. Minh B. Q., 2015 IQ-TREE: a fast and effective stochastic algorithm for estimating maximum-likelihood phylogenies. Mol. Biol. Evol. 32: 268–274. 25371430 10.1093/molbev/msu300 PMC4271533

[bib24] Nix D. A. Eisen M. B., 2005 GATA: a graphic alignment tool for comparative sequence analysis. BMC Bioinformatics 6: 9. 15655071 10.1186/1471-2105-6-9 PMC546196

[bib25] Simpson J. T. Wong K. Jackman S. D. Schein J. E. Jones S. J., 2009 ABySS: a parallel assembler for short read sequence data. Genome Res. 19: 1117–1123. 19251739 10.1101/gr.089532.108 PMC2694472

[bib26] Tsui C. K. M. DiGuistini S. Wang Y. Feau N. Dhillon B., 2013 Unequal recombination and evolution of the mating-type (*MAT*) loci in the pathogenic fungus *Grosmannia clavigera* and relatives. G3 3: 465–480. 23450093 10.1534/g3.112.004986 PMC3583454

[bib27] Wang S. Hu T. Wang Y. Luo Y. Michailides T. J., 2016 New understanding on infection processes of Valsa canker of apple in China. Eur. J. Plant Pathol. 146: 531–540.

[bib28] Wang, X., 2007 Pathogen of apple tree valsa canker in China: a combined analysis of phenotypic characteristics and rDNA-ITS sequences. Ph.D. Thesis, Northwest A&F University, Yangling.

[bib29] Wang X. Wei J. Huang L. Kang Z., 2011 Re-evaluation of pathogens causing Valsa canker on apple in China. Mycologia 103: 317–324. 21415290 10.3852/09-165

[bib30] Wang X. Zang R. Yin Z. Kang Z. Huang L., 2014 Delimiting cryptic pathogen species causing apple Valsa canker with multilocus data. Ecol. Evol. 4: 1369–1380. 24834333 10.1002/ece3.1030 PMC4020696

[bib31] Yin Z. Liu H. Li Z. Ke X. Dou D., 2015 Genome sequence of Valsa canker pathogens uncovers a potential adaptation of colonization of woody bark. New Phytol. 208: 1202–1216. 26137988 10.1111/nph.13544

